# Repertory of the Back

**Published:** 1898-09

**Authors:** E. H. Wilsey

**Affiliations:** Parkersburg, W. Va.


### REPERTORY OF THE BACK.

E. H. Wilsey, M. D., Parkersburg, W. Va.

(Continued from July number, page 315.)

### SCAPULÆ.

**Aching** beneath lower angles of scapula, Merc.
— between scapula and drawing, *Lyco.*
— and tension between scapula, *Nux.*
— and tensive between scapula while lying down and moving, Sul.
— and tensive between scapula and in sacrum, *Calc-ars.*
— between scapulæ, Æsculus, Ailanth.
— and burning along hips and scapula, also along spine, *Carbo-veg.*
— beneath lower tip of right scapula in P. M. with a bruised feeling, Calend.
— drawing aching pain in left scapula and joints of shoulder, Ptelia.
— in right scapula if he moves, *Cina.*
— in and pain mostly below scapula, Calc-phos.
— smarting and stinging in post-lateral margins of scapula, generally worse on left side, Zizia.
— under right scapula then between at 11 A. M., Merc.
— under point of left scapula upon lifting arm, Conval.
— under lower angle of right scapula, Conval.
**Above,** painful tension above the right scapula, Cicuta.

SCAPULÆ.

**Across** scapula, a tensive cutting, Rhus.
— scapula, a sharp cutting, *Glon.*
— pain across scapula extending down arms. *Ars.*
— pain in right scapula extending across shoulders to clavicle, worse by moving arm or head, better by pressure, Mag-m.
— stiff neck, also down between scapula and across shoulders, Lac-ac.
— dull boring, sticking in left scapula extending across spine, Menyan.
**Acromion,** pain at acromion process, left scapula, *Aspar.*
**Acute** pain under left scapula, Arundo.
**Afternoon,** stitches in upper part of left scapula in morning, with burning of skin on same place, Canth.
**Alternating** dull with aching and sharp pain beneath right scapula at its lower or inferior angle, Bry.
— pain along inner border of right scapula with heat and chills, alternating and extending to kidneys where there is heat and pain, Sang.
**Angle,** pain going along inner edges of left scapula, extending below inferior angle or through lower half of left side of thorax, *Ran-b.*
**Ants,** crawling as from ants or going to sleep in scapulæ, Anac.
**Beaten,** right scapula and small of back painful as if beaten, *Arn.*
**Bed,** pain in right scapula on lying in bed, Cepa.
**Behind,** fine, burning stitches in and behind right scapula, extending toward ribs, *Asaf.*
— right scapula, a pain with a feeling of a plug, at times throbbing as if it had been beaten, Kreos.
— left scapula, a pain, Iris-vers.
**Below,** pain intense below lower angle of right scapula through to chest, *Chen-a.*
— burning in skin below top of left shoulder, Lyco.
— clucking below left scapula, Lyco.

SCAPULÆ.

**Below,** pain below left scapula, with cough, *Sticta.*
— pain below left scapula, Aphis.
— pain, deep seated below point of scapulæ toward left, worse when sitting, especially when riding, Fluor-ac.
— drawing pressure below right scapula, now on the back, now on the side, especially sensible while sitting, when arm is held out from body, Sepia.
— dull stitches below right scapula, Zinc.
— pain below right scapula in evening after exertion, by deep inspiration, and by moving right arm, Ruta.
— scapulæ, a gnawing, tearing, shooting pain, Agari.
— right scapula, extending around to front of body, a very much inflamed spot about the size of the palm of the hand, painful to touch ; soon after pimples appear in a large group, causing violent burning, Cist-can.
— pressive pain in back below scapulæ, Calc-c.
— pressive pain in back below left scapula, Zinc.
— pressive drawing in spine below and between scapulæ, more violently when moving, especially when turning the body, Stann.
— pressive tension in back below right scapula down the back toward axilla, Zinc.
— pressure and shooting below left scapula, with sensitiveness when pressing upon it, Nat-c.
— pressure in back below scapulæ, Carduus, Lyco.
— pressure close below scapulæ, Lyco.
— pressure, pain below right scapula, better by pressure and lying down, especially on right side, Ruta.
— pressure, wandering pain in dorsal muscles below scapulæ, Brom.
— pain below right scapula, more toward spine after sitting a long time, Cepa.
— pain, as if something were sticking below right scapula, increased at first and disappearing after three hours' sleep, Ars-hyd.

### SCAPULÆ.

**Below,** pain below right scapula, extends over a spot as large as the palm of the hand, Ruta.
— stinging below right scapula, Lyssin.
— stitch below left scapula when drawing the scapula inward, not when breathing, Nat-mur.
— frequent stitches for a quarter of an hour below both scapulæ, Phos.
— stitch below left scapula, extending into left chest, Zinc.
— pain below and in region of right scapula, Physos.
— a sudden pain below left scapula, Nat-sul.
— tearing below right scapula, Dig.
— tearing pain on inner side of scapula and below it on bending body back and to the left, Aurum-fol.
— tensive pain in right side of back below scapulæ, especially when lying on left side, Sepia.
— pain below right scapula, when severe it extends to the corresponding part of left side, Ruta.
— pains and aches in and near, between and mostly below shoulder-blades; pulsating, throbbing, jerking, Calc-phos.
— severe muscular twitching below left scapula, Mez.
— dull twitching, shooting just below and beside left scapula, Zinc.
**Beneath** left scapula, a sticking drawing when sitting, Millef.
— scapula, a bubbling sensation also in back and upper arm, Squilla.
— a bubbling sensation beneath left scapula, Lyco.
— a sticking beneath left scapula in evening, Kali-nit.
— left scapula a burning, Can-sat.
— and near left scapula a dull jerking, Zinc.
— right scapula at night a sticking, Kali-nit.
— left scapula a pressure in back, Zinc.
— right scapula, a pressing tension in back, extending toward axilla, Zinc.
— sticking behind and beneath tip of right scapula, impeding respiration, Clematis.

SCAPULÆ.

**Beneath** right scapula, a drawing, with pressure, impeding respiration, Rhus.
— right scapula, stitches, *Chelid.*
— and between scapulæ, a sticking, Colchi.
— right scapula, a sticking, extending to small of back and head, Lyco.
— right scapula, a pulsating sticking when sitting, Sambu.
— stitches beneath scapulæ which take away the breath and do not permit stooping, Sul.
**Between** scapulæ a drawing aching, *Lyco.*
— scapulæ, a great aching, and under left scapula, extending to left lung, worse on expiration, Sepia.
— aching, Æscul., Ailanth.
— scapulæ and sacrum, a violent ache, *Calc-ars.*
— a dull, boring sticking in left scapula, extending across scapulæ, Meny.
— boil between scapulæ, large, *Iod.*
— breath, stitches between scapulæ on drawing a long breath, Prun-sp.
— breath, stitches between scapulæ, as with knives, when lying on back, waking her and causing short breath, better by lying on right scapula, K-nit.
— breathing, sticking between scapulæ on deep breathing, Prun-sp., Acon.
— breathing, stitches between scapulæ and fore part of chest impeding, more when stooping than when sitting quietly, Nit-ac.
— boring between scapulæ, Phos-ac.
— breathing, pain between scapulæ, extending downward, especially on deep breathing, Meny.
— scapulæ, a bruised pain when breathing, Aco.
— bruised feeling between scapulæ, with burning, Mag-m.
— bruised pain between scapulæ, Amm-m.
— burning sensation between scapulæ before vomiting, *Rhus.*
— burning and tingling between scapulæ, Sabad.

### SCAPULÆ.

**Between,** burning, as from hot coals between scapulæ, worse in summer, *Lyco.*

— burning and soreness between scapulæ, Phos-ac.

— burning between scapulæ, *Glon.*, *Phos.*, Sul.

— burning and heat in dorsal region mostly between lower half of scapulæ while sitting reading at night, *Helonias.*

— corrosive burning between shoulders, under the right shoulder joint on the sacrum and on the nates in the evening after lying down, Sul.

— burning and tearing between spine and right scapula, Zinc.

— burning cutting between shoulders with burning as if it would be cut through there, Sul-ac.

— first a pressure between scapulæ, then from there a burning extending to the crest of the ilium, equally while at rest and in motion, also felt when the hand is laid upon it, Sul.

— chill, as if a piece of ice were lying between scapulæ and followed by a chill with goose-flesh all over, Lachn.

— chill begins between scapulæ, *Rhus.*

— chills like streams of cold water running down from between scapulæ to sacral region, Variol.

— chilliness between scapulæ, *Caps.*

— pain between scapulæ and on chest, Lach.

— tension between scapulæ on taking off coat, Nat-c.

— as if cold wind were blowing on back between scapulæ, Caustic.

— cold perspiration, skin dry and cool, sensation between scapulæ as if wet with a, Lachnanthes.

— cold, sensation as of an icy cold hand between scapulæ, *Sepia.*

— scapulæ, cold sensation as if touched by ice, Agari.

— coldness between scapulæ, *Nat-c.*, Viola-tri.

— coldness, as from a piece of ice between scapulæ, better by heat, *Puls.*

— coldness between scapulæ and in back not relieved by feather or wool covering, followed by itching, *Amm-m.*

— colic, pain between scapulæ, with *Amm-c.*

### SCAPULÆ.

**Between** contractive, pain between scapulæ, Graph., *Guaicum.*
— scapulæ, contractive pain when standing, *China.*
— constrictive pain between scapulæ, *Merc-cy.*
— constriction between scapula, Nux-v.
— constriction between scapulæ, hindering motion of arms and respiration, Thuja.
— a pain between scapulæ when coughing, Stram.
— sticking between scapulæ on taking off corset, Lauroc.
— cramp between scapulæ during motion, *Ip.*
— cramp between scapulæ, with palpitation, *Phos.*
— cutting between scapulæ, *Hyperi.*, Meny.
— cutting pain between scapulæ while resting, Calc-c.
— cutting pain between scapulæ, passing through to sternum, with sensation of pressure or constriction to chest, in afternoon, *Lac-c.*
— cutting, violent pain between scapulæ, Kali-nit.
— cutting, between scapulæ, then about last dorsal vertebræ, Mer-i-fl.
— cutting between shoulders with burning as if it would be cut through there, Sul-ac.
— distress between scapulæ, *Phos.*
— an uneasy dragging between scapulæ in spine, Naja.
— a drawing pressing in spine between scapulæ, worse on motion, Stann.
— scapulæ, a drawing, *Hepar*, *Lyco.*, *Ars.*, Calc-c., Calend., Guaj., Nat-c., Puls., Thuja, Viola-tr., Zinc.
— drawing between scapulæ in evening, *Lyco.*
— drawing downward between scapulæ, Gratiola.
— scapulæ, a drawing pain, necessitating lying down, *Ars.*
— scapulæ, a drawing pain, *Calc-c.*, Diosc.
— pain between scapulæ, drawing to small of back, *Drosera.*
— drawing between scapulæ at night during menses, better by bending backward, Sil.
— drawing between scapulæ, compelling him to lie down, *Ars.*
— drawing between scapulæ and in upper part of chest, Sepia.

SCAPULÆ.

**Between,** drawing between scapulæ, tensive at 6 P. M., Kali-nit.
— drawing and rigidity between scapulæ in open air, extending down as far as the anus in paroxysms, and terminating in a stitch when lying or sitting, Nat-carb.
— drawing stitches between scapulæ, extending into chest when moving arms, *Camphor*.
— pain between scapulæ in drunkards and with suppressed menses, *Ars*.
— eructation, stitches in and between scapulæ, always followed by eructation, Nit-acid.
— eruption between scapulæ, Coccul.
— fall, a spasmodic sensation between scapulæ when walking, as if he would fall headlong, Calad.

